# Magnetoelastic Coupling and Delta-E Effect in Magnetoelectric Torsion Mode Resonators

**DOI:** 10.3390/s21062022

**Published:** 2021-03-12

**Authors:** Benjamin Spetzler, Elizaveta V. Golubeva, Ron-Marco Friedrich, Sebastian Zabel, Christine Kirchhof, Dirk Meyners, Jeffrey McCord, Franz Faupel

**Affiliations:** Institute of Materials Science, Faculty of Engineering, Kiel University, 24143 Kiel, Germany; besp@tf.uni-kiel.de (B.S.); elgo@tf.uni-kiel.de (E.V.G.); rmfr@tf.uni-kiel.de (R.-M.F.); seza@tf.uni-kiel.de (S.Z.); cki@tf.uni-kiel.de (C.K.); dm@tf.uni-kiel.de (D.M.); jmc@tf.uni-kiel.de (J.M.)

**Keywords:** delta-E effect, magnetoelectric, magnetoelastic, resonator, torsion mode, bending mode, magnetic modeling, MEMS, FEM

## Abstract

Magnetoelectric resonators have been studied for the detection of small amplitude and low frequency magnetic fields via the delta-E effect, mainly in fundamental bending or bulk resonance modes. Here, we present an experimental and theoretical investigation of magnetoelectric thin-film cantilevers that can be operated in bending modes (BMs) and torsion modes (TMs) as a magnetic field sensor. A magnetoelastic macrospin model is combined with an electromechanical finite element model and a general description of the delta-E effect of all stiffness tensor components *C_ij_* is derived. Simulations confirm quantitatively that the delta-E effect of the *C*_66_ component has the promising potential of significantly increasing the magnetic sensitivity and the maximum normalized frequency change $\Delta f_{r}$. However, the electrical excitation of TMs remains challenging and is found to significantly diminish the gain in sensitivity. Experiments reveal the dependency of the sensitivity and $\Delta f_{r}$ of TMs on the mode number, which differs fundamentally from BMs and is well explained by our model. Because the contribution of *C*_11_ to the TMs increases with the mode number, the first-order TM yields the highest magnetic sensitivity. Overall, general insights are gained for the design of high-sensitivity delta-E effect sensors, as well as for frequency tunable devices based on the delta-E effect.

## 1. Introduction

In recent years, thin-film magnetoelectric sensors have been studied, frequently envisioning biomedical applications in the future [1,2]. Such applications often require the measurement of small amplitude and low frequency magnetic fields [1,2,3]. With the direct magnetoelectric effect, such small detection limits are only obtained at high frequencies and in small-signal bandwidths of a few Hz [2,4]. One way to overcome these limitations is by using a modulation scheme based on the delta-E effect. The delta-E effect is the change of the effective elastic properties with magnetization due to magnetoelastic coupling [5,6,7,8]. It results from inverse magnetostriction that adds additional stress-induced magnetostrictive strain to the purely elastic Hookean strain. The delta-E effect can occur generally in various elastic moduli and several components of the elastic stiffness tensor C [9,10]. Hence, it is sometimes referred to as the delta-C effect [11]. Typically, delta-E effect sensors are based on magnetoelectric resonators that are electrically excited via the piezoelectric layer at or close to the resonance frequency $f_{r}$. Upon the application of a magnetic field, the magnetization changes and the delta-E effect alters the mechanical stiffness tensor of the magnetostrictive layer. If the altered stiffness tensor components contribute to the resonance frequency of the excited mode, the resonance frequency changes, which can be read-out electrically. The delta-E effect of the Young’s modulus has especially been studied thoroughly in soft magnetic amorphous materials [12,13,14,15,16,17,18]. It was used for magnetic field sensing with magnetoelectric plate resonators [19,20,21,22] and beam structures [23,24,25,26,27,28,29,30,31,32]. Such resonators are operated in bending or bulk modes and some have achieved limits of detection down to the sub-nT regime at low frequencies. Microelectromechanical systems (MEMS) cantilever sensors based on the delta-E effect were recently used for the mapping of magnetically labeled cells [33], and have shown promising properties for sensor array applications [34].

In contrast to the delta-E effect of the Young’s modulus, the delta-E effect of the shear modulus has been studied less extensively [35] and mainly in amorphous wires [36,37]. It has been used for a different kind of delta-E effect sensors where shear waves, traveling through the magnetoelastic material, are influenced by the delta-E effect. This concept was realized with bulk acoustic shear waves in amorphous ribbons [38] and recently with surface acoustic shear waves in magnetic thin film devices [10,39,40,41,42]. Only very few studies investigate torsion modes in beam structures [43,44], either with electrostatically actuated cantilevers [43] or double-clamped beams [44]. Both studies are limited to specific configurations of the magnetic system and consider neither the full tensor relations of the mechanics and the delta-E effect nor higher resonance modes. Until now, a comprehensive experimental and theoretical analysis has been missing as well as a discussion of implications for the design of delta-E effect-based devices. 

## 2. MEMS Torsion Mode Sensors

**Figure 1:**
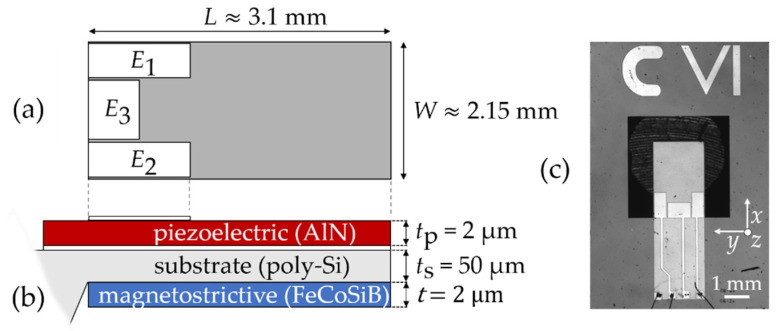
Delta-E effect sensor analyzed in this study: (**a**) schematic top view of the cantilever, with three different electrodes $E_{1}$, $E_{2}$ and $E_{3}$ of lengths $L_{1}=L_{2}\approx 1\;\mathrm{mm}$, $L_{3}\approx 0.6\;\mathrm{mm}$ and widths $W_{1}=W_{2}\approx 0.5\;\mathrm{mm}$ and $W_{3}\approx 1\;\mathrm{mm}$; (**b**) schematic side-view of the cantilever with the thickness of the functional layers and the poly-Si substrate; (**c**) top-view photograph of the fabricated structure.

In this study, all measurements and models are made for a microelectromechanical system (MEMS) technology-fabricated cantilever with an electrode design that permits the excitation of torsion modes. A sketch including dimensions and layer structure and a top-view photograph of the design are shown in Figure 1. The approximately 3.1 mm-long and 2.15 mm-wide cantilever consists of a ≈ 2 µm-thick piezoelectric layer of AlN [45] on a 50 µm-thick poly-Si substrate. A 2 µm-thick amorphous magnetostrictive multilayer is deposited on the rear side. A magnetic field is applied during the deposition to induce a magnetic easy axis along the short cantilever axis. For actuation and read-out, three top electrodes ($E_{1}$, $E_{2}$ and $E_{3}$) of 100 nm-thick Au with lengths $L_{1}=L_{2}\approx 1\;\mathrm{mm}$ and $L_{3}\approx 0.6\;\mathrm{mm}$ and widths $W_{1}=W_{2}\approx 0.5\;\mathrm{mm}$ and $W_{3}\approx 1\;\mathrm{mm}$ contact the AlN layer on the top. The counter electrode (150 nm Pt) covers the whole beam area and is located between the AlN layer and the substrate. All measurements are performed with electrode $E_{1}$. As a magnetostrictive material, we use a 2 µm multilayer of $20\times (100\;\mathrm{nm}\;{\left({\mathrm{Fe}}_{90}{\mathrm{Co}}_{10}\right)}_{78}{\mathrm{Si}}_{12}B_{10}$ and 6 nm Cr). It is covered by a top Cr-layer that serves as a protection against corrosion. More information about the layer structures and the fabrication process can be found elsewhere [27]. In contrast to the sensors in Ref. [27], the sensor presented here is significantly wider and the adapted electrode design additionally permits the excitation of torsion modes. Details on the geometry are given in the appendix.

## 3. Sensitivity

### 3.1. Definition of the Sensitivity

An important parameter that characterizes a magnetic field sensor is its sensitivity. During sensor operation, an alternating voltage is applied to excite the cantilever at its mechanical resonance frequency $f_{r}$. Applying a magnetic field, shifts $f_{r}$ via the delta-E effect and correspondingly the sensor’s admittance characteristic on its frequency axis *f*. Hence, the magnitude |Y|=abs{Y} and phase angle ϕ=arg{Y} of the sensor admittance Y depend on the magnetic field. Consequently, the ac magnetic field to be measured causes an amplitude modulation (am) and phase modulation (pm) of the current through the sensor. Detailed information on the operation and read-out can be found elsewhere [46,47,48]. The linearized change of |Y| and ϕ with the magnetic field can be described by the amplitude sensitivity $S_{\mathrm{am}}=S_{Y,r}\cdot S_{H,r}$ and the phase sensitivity $S_{\mathrm{pm}}=S_{\phi ,r}\cdot S_{H,r}$ [49], respectively. Both sensitivities have a magnetic part $S_{H,r}$ that includes the delta-E effect and an electric part $S_{Y,r}$ or $S_{\phi ,r}$, which can be determined from the admittance. We refer to the three sensitivities as relative sensitivities, because they are normalized to the excitation frequency $f_{\mathrm{ex}}=f_{r}$. The normalization is required to eventually compare the electrical and magnetic sensitivities of sensors with different geometries operated at different $f_{r}$ or in different resonance modes. Usually a magnetic bias field $H_{0}$ is applied to operate the sensor at optimum conditions. The relative sensitivities are then defined in linear approximation as derivatives [49]:

(1) $$S_{Y,r}≔{\frac{\partial \left|Y\right|}{\partial f}|}_{f=f_{r},\;H=H_{0}}\cdot f_{r};\;\;\;\;\;\;S_{\phi ,r}≔{\frac{\partial \phi }{\partial f}|}_{f=f_{r},H=H_{0}}\cdot f_{r};\;\;\;\;\;\;\;S_{H,r}≔\frac{1}{f_{r}}{\frac{\partial f_{r}}{\partial \mu_{0}H}|}_{H=H_{0}}\;\;,$$

with the magnetic vacuum permeability $\mu_{0}\approx 4\pi \cdot 10^{-7}\;N/A^{2}$. From Equation (1), the relative magnetic sensitivity $S_{H,r}$ is the linearized and normalized change of the resonance frequency $f_{r}$ with the applied magnetic flux density $\mu_{0}H$. 

### 3.2. Magnetic Sensitivity of Arbitrary Resonance Modes

The delta-E effect is included in the relative magnetic sensitivity $S_{H,r}$ because the resonance frequency $f_{r}=f_{r}\left(C_{ij}\right)$ is a function of the stiffness tensor components $C_{ij}$. Depending on the respective resonance mode, different $C_{ij}$ dominate $f_{r}$ and depending on the magnetoelastic properties they might result in non-zero $S_{H,r}$. To describe $S_{H,r}$ for arbitrary resonance modes, it can be separated into a purely mechanical part $f_{r}^{-1}\partial f_{r}/\partial C_{ij}$ that contains the resonance properties of the structure and a purely magnetoelastic part $\partial C_{ij}/\partial \mu_{0}H$:

(2) $$S_{H,r}≔\frac{1}{f_{r}}{\frac{\partial f_{r}}{\partial \mu_{0}H}|}_{H=H_{0}}=\sum_{i=1}^{3}\sum_{j=1}^{3}\frac{1}{f_{r}}\frac{\partial f_{r}}{\partial C_{ij}}\;\;{\frac{\partial C_{ij}}{\partial \mu_{0}H}|}_{H=H_{0}}≔\sum_{i=1}^{3}\sum_{j=1}^{3}\partial_{C}f_{r,ij}\;\partial_{H}C_{ij}.$$

If treated separately, the factors $\partial f_{r}/\partial C_{ij}$ and $\partial C_{ij}/\partial \mu_{0}H$ must be normalized to remove the dependency on the absolute value of $C_{ij}$ that cancels out in $S_{H,r}$. We define:

(3) $$\partial_{C}f_{r,ij}≔{\frac{C_{ij}}{f_{r}}\frac{\partial f_{r}}{\partial C_{ij}}|}_{\;H=H_{0}};\;\;\;\;\;\;\partial_{H}C_{ij}≔\frac{1}{C_{ij}}{\frac{\partial C_{ij}}{\partial \mu_{0}H}|}_{H=H_{0}}.$$

From Equation (3), the factor $\partial_{C}f_{r,ij}$ represents a normalized measure for the influence of the stiffness tensor component $C_{ij}$ on the resonance frequency $f_{r}$ of the considered resonance mode. It is a purely mechanical quantity and hence determined by the geometry, the resonance mode, and the effective mechanical properties of the resonator. The second factor $\partial_{H}C_{ij}$, includes the delta-E effect and describes the normalized influence of the applied flux density $\mu_{0}H$ on $C_{ij}$. Hence, the two factors quantify the mechanical and the magnetoelastic parts of the relative magnetic sensitivity $S_{H,r}$. They will be used later to analyze the sensitivity and the frequency detuning of higher bending and torsion modes of the cantilever. 

## 4. Sensor Modelling

The model used to describe and analyze the sensor consists of two parts. With a semi-analytical magnetoelastic macrospin model, the delta-E effect is obtained, i.e., the effective mechanical stiffness tensor ***C***(*H*) as a function of the applied field *H*. It is used as an input for an electromechanical finite element mechanics (FEM) model that describes the resonance frequency and the sensor’s impedance response. In addition to a macrospin approximation, we assume a quasi-static magnetization behavior. Consequently, it is only valid for operation frequencies and magnetic field frequencies far below the ferromagnetic resonance frequency (FMR). The FMR generally depends on the geometry and the magnetic properties of the thin-film [50] and can cause a frequency dependency of the delta-E effect [51]. For the soft-magnetic material and thin-film geometry used here, it is in the GHz regime [51,52]. Because the operation frequencies are of the order of several kHz, magnetodynamic effects and the frequency dependency of the delta-E effect are neglected [51]. Due to the low frequencies, we assume that also electrodynamic effects can be omitted in the electromechanical model. In the following, both parts of the model are discussed in detail.

### 4.1. Electromechanical Model

**Figure 2:**
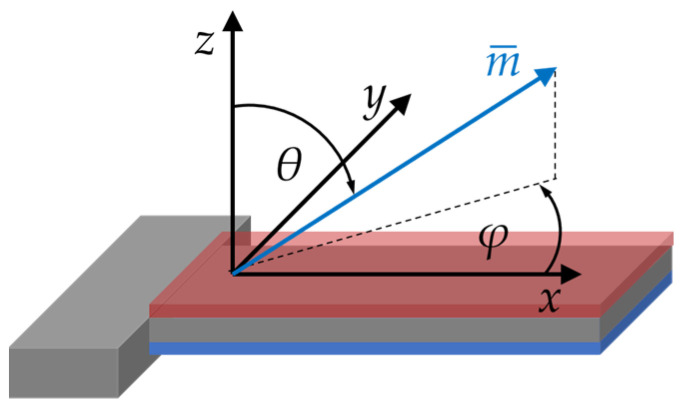
Coordinate system used for the electromechanical and the magnetic model. All three components $m_{i}$ of the reduced magnetization vector $\bar{m}$ are described by the polar angle θ and the azimuthal angle φ.

In the electromechanical part of the model, we consider a simplified cantilever geometry, reduced to the poly-Si substrate, the piezoelectric AlN layer, and the magnetic FeCoSiB layer. Details on the geometry used are given in Appendix C. We assume all materials to be mechanically linear, which is a good approximation at sufficiently small excitation voltages. The material parameters used are given in the appendix. The cantilever is oriented in a cartesian coordinate system as illustrated in Figure 2, used throughout this paper. The mechanical equation of motion is given by (e.g., [53])

(4) $$\rho \frac{\partial^{2}\bar{u}}{\partial t^{2}}=\bar{\nabla }\cdot \sigma \;,\;$$

if no external forces are present. It includes the displacement vector $\bar{u}$, the time *t*, the mass density ρ, and the divergence $\bar{\nabla }\cdot \sigma$ of the mechanical stress tensor σ. For sufficiently small excitation frequencies, eddy current effects can be neglected and the electrostatic equations [54]:

(5) $$\begin{array}{c} \bar{E}=-\bar{\nabla }U,\\ \bar{\nabla }\cdot \bar{D}=\rho_{c}, \end{array}$$

are valid in good approximation. They include the electrical vector field $\bar{E}$, the gradient $\bar{\nabla }U$ of the electrical potential U and the divergence $\bar{\nabla }\cdot \bar{D}$ of the electric flux density $\bar{D}$ with the free charge density $\rho_{c}$. The electrostatic equations are coupled to the mechanical equation of motion via the constitutive piezoelectric equations, here in the stress-charge form [54,55]:

(6) $$\begin{array}{c} \sigma =C^{*}\varepsilon -e_{c}^{T}\bar{E}\\ \bar{D}=e_{c}\varepsilon -\varepsilon_{\mathrm{el}}\bar{E}\;, \end{array}$$

with the linear strain tensor ε and the complex mechanical stiffness tensor $C^{*}=C\left(1+i\eta \right)$. Its real part is the material’s stiffness tensor C and its imaginary part Cη includes the isotropic loss factor η, which is used to consider damping in the materials [56]. The electromechanical coupling tensor is denoted as $e_{c}$ and the electrical permittivity tensor as $\varepsilon_{\mathrm{el}}$. For the calculation, we set fixed boundary conditions $\left(\bar{u}=0\right)$ at the left face of the beam. For the piezoelectric material, we assumed at the boundaries $\bar{n}\bar{D}=0$ (with surface normal vector $\bar{n}$), and an initial value for the electric potential of U=0, except for the area covered by the electrodes. The electrodes are modeled with a fixed potential boundary condition, where an alternating voltage $U_{\mathrm{app}}=U_{0}\cdot \exp\left(i\left[\omega t+\varphi_{v}\right]\right)$ is applied, with amplitude $U_{0}$, the angular frequency ω and phase angle $\varphi_{v}$. To calculate the electrical admittance Y=I/U the current I is obtained from integrating the surface charge density over the electrode areas. For the solution, a linear response of the system is assumed, with a displacement of the form $\bar{u}=\hat{u}\cdot \exp\left(i\left[\omega t+\varphi_{u}\right]\right)$ and a solution for the electrical potential of $U=\hat{U}\cdot \exp\left(i\left[\omega t+\varphi_{v}\right]\right)$. The equations are solved within a frequency domain study in ${\mathrm{COMSOL}}^{®}$ Multiphysics v. 5.3a (COMSOL AB, Stockholm, Sweden) [56]. All material parameters used are given in the appendix.

### 4.2. Magnetoelastic Model

For the magnetic model, we consider the enthalpy density function of a macrospin with a uniaxial anisotropy energy density, an external magnetic field, a demagnetizing term, and magnetoelastic energy density. Using Einstein’s summation convention, the enthalpy density term we use is:

(7) $$u=K\left(1-{\left(m_{i}EA_{i}\right)}^{2}\right)-\mu_{0}M_{s}m_{i}H_{i}-\frac{1}{2}\mu_{0}M_{s}m_{i}H_{d,i}-\sigma_{j}\lambda_{j}\;\;\;\;\;\mathrm{with}\;i=1,2,3\;\;\;j=1,\ldots ,6\;.$$

In this equation, the components of the reduced magnetization vector are denoted by $m_{i}$, the magnitude of the magnetization vector by $M_{s}$ and the magnetic vacuum permeability by $\mu_{0}$. The effective easy axis of magnetization is characterized by its orientation vector $EA_{i}$ and the effective first-order uniaxial anisotropy energy density constant K. The components of the external magnetic field vector are given by $H_{i}$ and the components of the mean demagnetizing field by $H_{d,i}=-D_{ii}m_{i}M_{s}$, with the main diagonal components $D_{ii}$ of the demagnetizing tensor. For the magnetoelastic energy density, we use the coupling term $-\sigma_{i}\lambda_{i}$ with the stress tensor components $\sigma_{i}$ and the components $\lambda_{i}$ of the isotropic magnetostrictive strain tensor. Both are given in Voigt’s notation. The coupling term results from omitting magnetostrictive self-energy and incorporating the term constant with stress into *K* [57]. In the following, the polar angle θ and the azimuthal angle φ of $\bar{m}$ in the spherical coordinate system (Figure 2) are used to define its components $m_{i}$. The exact definition of all vector and tensor components is given in the appendix. The linearized change of the elastic compliance components $S_{ij}$ with the magnetic field and stress is derived from the expression

(8) $$S_{ij}\left(H,\sigma \right)=\frac{\partial \varepsilon_{i}}{\partial \sigma_{j}}=\frac{\partial \left(e_{i}+\lambda_{i}\right)}{\partial \sigma_{j}}≔S_{m,ij}+\Delta S_{ij}\;.$$

where the first summand $S_{m,ij}$ is the constant, fixed magnetization elastic compliance tensor component. The magnetization dependent part $\Delta S_{ij}$ can be obtained from the equilibrium conditions that are given by the first-order derivatives of u:

(9) $$u_{\varphi }≔\frac{\partial u}{\partial \varphi }=0\;\;\;\mathrm{and}\;u_{\theta }≔\frac{\partial u}{\partial \theta }=0\;.\;$$

From these equilibrium conditions a general expression for the linearized change $\Delta S_{ij}$ of the compliance tensor can be derived (Appendix A). Denoting the second-order derivatives as $u_{\varphi \varphi }$ and $u_{\theta \theta }$ it is:

(10) $$\Delta S_{ij}≔\frac{\partial \lambda_{i}}{\partial \sigma_{j}}=-\frac{\partial \lambda_{i}}{\partial \varphi }\frac{\partial u_{\varphi }}{\partial \sigma_{j}}\frac{1}{u_{\varphi \varphi }}-\frac{\partial \lambda_{i}}{\partial \theta }\frac{\partial u_{\theta }}{\partial \sigma_{j}}\frac{1}{u_{\theta \theta }}\;.$$

This expression permits a quick calculation of the compliance tensor for different magnetic systems described by an enthalpy density u. From Equation (10), the non-zero components of ΔS for in-plane magnetization (θ=π/2) are:

(11) $$\Delta S_{11}=\Delta S_{22}=-\Delta S_{12}=\frac{9\lambda_{s}^{2}\cos{\left[\varphi \right]}^{2}\sin{\left[\varphi \right]}^{2}}{u_{\varphi \varphi }}\;,$$

(12) $$\Delta S_{16}=-\Delta S_{26}=-\frac{9\lambda_{s}^{2}\cos\left[\varphi \right]\cos\left[2\varphi \right]\sin\left[\varphi \right]}{u_{\varphi \varphi }}\;,$$

(13) $$\Delta S_{44}=\frac{9\lambda_{s}^{2}\sin{\left[\varphi \right]}^{2}}{u_{\theta \theta }}\;,$$

(14) $$\Delta S_{45}=\frac{9\lambda_{s}^{2}\cos\left[\varphi \right]\sin\left[\varphi \right]}{u_{\theta \theta }}\;,$$

(15) $$\Delta S_{55}=\frac{9\lambda_{s}^{2}\cos{\left[\varphi \right]}^{2}}{u_{\theta \theta }}\;,$$

(16) $$\Delta S_{66}=\frac{9\lambda_{s}^{2}\cos{\left[2\;\varphi \right]}^{2}}{u_{\varphi \varphi }}\;.$$

The final compliance tensor for in-plane magnetization as a function of magnetic field and stress is:

(17) $$S(H,\sigma )=\left[\begin{array}{cccccc} S_{11} & S_{12} & S_{m,13} & 0 & 0 & \Delta S_{16}\\ S_{12} & S_{22} & S_{m,23} & 0 & 0 & \Delta S_{26}\\ S_{m,13} & S_{m,23} & S_{m,33} & 0 & 0 & 0\\ 0 & 0 & 0 & S_{44} & \Delta S_{45} & 0\\ 0 & 0 & 0 & \Delta S_{45} & S_{55} & 0\\ \Delta S_{16} & \Delta S_{26} & 0 & 0 & 0 & S_{66} \end{array}\right]\;\mathrm{with}\;S_{ij}(H,\sigma )=S_{m,ij}+\Delta S_{ij}.$$

Because in our case both, $S_{m}$ and ΔS. are symmetric, and S is also symmetric. Note that $S_{m,16}=S_{m,26}=S_{m,45}=0$ in our isotropic magnetic material and consequently $S_{16}=\Delta S_{16}$, $S_{26}=\Delta S_{26}$ and $S_{45}=\Delta S_{45}$. Finally, the stiffness tensor C is obtained by numerically calculating the inverse $C\left(H,\sigma \right)=S{\left(H,\sigma \right)}^{-1}$. It has the same non-zero components and symmetry. All equations (Equations (11)–(17) are obtained from Equation (10) assuming in-plane magnetization (θ=π/2) and are valid for the isotropic magnetoelastic coupling used in the enthalpy density function (Equation (7)). For all the following simulations, we additionally assume in-plane magnetic fields ($\theta_{H}=\pi /2)$ and an in-plane easy axis ($\theta_{\mathrm{EA}}=\pi /2)$.

These two assumptions influence and simplify *u* and its derivatives, which are given in the appendix.

## 5. Implications of the Magnetic Model

In the following, results for the $C_{ij}$ of the magnetoelastic model are discussed at the example of a thin-film geometry. For the calculations, we assumed zero static stress ($\sigma_{i}=0$) and $D_{33}=1$. The large shape anisotropy results in $C_{44}\approx C_{m,44}$ and $C_{55}\approx C_{m,55}$. We limit the discussion to the $C_{11}$, $C_{12}$ and $C_{66}$ components as they are most relevant for torsion and bending modes.

**Figure 3:**
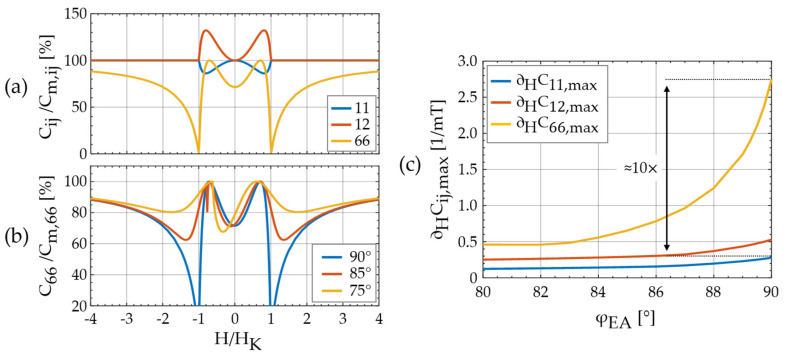
(**a**) Magnetic field dependent components $C_{ij}$ of the effective stiffness tensor for an ideal hard axis magnetization process of a macrospin. The external magnetic field with magnitude H is applied along the x axis and normalized the anisotropy field *H*_K_; (**b**) component $C_{66}$ for different angles $\varphi_{\mathrm{EA}}$ of the magnetic easy axis to the x axis; (**c**) maximum value $\partial_{H}C_{ij,\max}$ of $\partial_{H}C_{ij}\left(H\right)$ (Equation (3)) for the $C_{11}$ and $C_{66}$ components as functions of the easy axis angle $\varphi_{\mathrm{EA}}$. For the calculations in (c), a distribution of effective anisotropy energy density is used as in Ref. [49] with a standard deviation of $\delta_{K}=15\;\%$ for a more quantitative estimation and to prevent singularities.

In Figure 3a, the normalized $C_{11}$, $C_{12}$ and $C_{66}$ components are plotted for a macrospin and $\varphi_{\mathrm{EA}}=90{}^{\circ}$. Because $u_{\varphi \varphi }\left(H=H_{K}\right)=0$ and so $\Delta S_{66}\left(H\to H_{K}\right)\to \infty$ (Equation (4)) it is $C_{66}\left(H=H_{K}\right)=0$. At $\left|H\right|>\left|H_{K}\right|$, it is $C_{66}<C_{m,66}$ with $C_{66}=C_{m,66}$ only for H→∞. Hence, for finite H even a small shear stress $\sigma_{6}$ can always tilt the magnetization vector out of the applied magnetic field direction. It occurs, because the magnetoelastic energy density contribution $-\sigma_{6}\lambda_{6}$ of the shear stress $\sigma_{6}$ is asymmetric around φ=0°. Its minimum is shifted by 45° compared to the minimum of the one-component at φ=0°. Consequently, at the two local maxima it is $C_{66}\left(\varphi =45{}^{\circ},135{}^{\circ}\right)=C_{m,66}$. The $C_{11}$ component shows two distinct minima but unlike the delta-E effect in the Young’s modulus (e.g., [6,14,49]) no discontinuities at $\left|H\right|=\left|H_{K}\right|$. Although the discontinuities are present in $S_{11}$ (not shown), they vanish during the inversion due to contributions of other $S_{ij}$ components to $C_{11}$. In contrast to $C_{11}$, $C_{12}$ stiffens with applied magnetic bias field because $\Delta S_{12}=-\Delta S_{11}$. The signs are a direct consequence of the positive isotropic magnetoelastic coupling. As the macrospin rotates towards the x axis, magnetostrictive expansion occurs along the x axis, but contraction occurs along the y axis. Compared with $C_{11}$, the maximum relative change of $C_{12}$ is larger because $S_{m,12}<S_{m,11}$, which results in a different weighting in Equation (8). In Figure 3b, $C_{66}$ is shown for three different angles of the easy axis $\varphi_{\mathrm{EA}}=90{}^{\circ},85{}^{\circ},$ and 75°. It is apparent that a change of $\varphi_{\mathrm{EA}}$ strongly influences $C_{66}$. Relative to $\varphi_{\mathrm{EA}}=90{}^{\circ}$, the two minima at $H=\pm H_{K}$ shift to a larger |H| and the minimum value increases strongly by more than 85% at $\varphi_{\mathrm{EA}}=85{}^{\circ}$ and about 95% at $\varphi_{\mathrm{EA}}=75{}^{\circ}$. The center minimum shifts due to the single domain hysteresis and decreases slightly with decreasing $\varphi_{\mathrm{EA}}$. A singularity occurs at $\varphi_{\mathrm{EA}}=85{}^{\circ}$ due to the magnetic discontinuity at the switching field of the single-spin model. Due to the strong impact of small deviations from $\varphi_{\mathrm{EA}}=90{}^{\circ}$ on $C_{66}\left(H\right)$, the magnetic sensitivity is expected to change notably with $\varphi_{\mathrm{EA}}$.

In the following, we quantify the influence of the $C_{ij}$ components on the relative magnetic sensitivity $S_{H,r}$ using $\partial_{H}C_{ij}$ as defined in Equation (3). Calculating $\partial_{H}C_{ij}$ requires forming the derivate $\partial C_{ij}/\partial H$, which results in singularities for the 66-component at $\varphi_{\mathrm{EA}}=90{}^{\circ}$ and |*H|* = |*H*_K_|. A finite derivative can be estimated by including the distribution of the effective anisotropy energy density *K* in a mean-field approach [15,49,58]. With such a distribution, inhomogeneities in the magnetization response are considered that can occur, e.g., from spatially varying stress or internal stray fields. We use a normal distribution of K with a standard deviation $\delta_{K}=15\;\%$ as a representative example value that has been used previously for a similar device [49]. We calculate $\partial_{H}C_{ij}\left(H\right)$ numerically from $C_{ij}\left(H\right)$ and extract the maximum $\partial_{H}C_{ij,\max}\left(H\right)$ for H>0, at various angles $\varphi_{\mathrm{EA}}$ of the easy axis. They are plotted in Figure 3c. As a result of the distribution, both $\partial_{H}C_{ij,\max}$ are finite at $\varphi_{\mathrm{EA}}=90{}^{\circ}$ with $\partial_{H}C_{66,\max}\approx 10\times \partial_{H}C_{11,\max}\approx 4.5\times \partial_{H}C_{12,\max}$. This is reduced to $\partial_{H}C_{66,\max}\approx 4\times \partial_{H}C_{11,\max}\approx 2\times \partial_{H}C_{12,\max}$ at $\varphi_{\mathrm{EA}}=80{}^{\circ}$. In conclusion, the $C_{66}$ component potentially offers a significantly larger magnetic sensitivity than the $C_{11}$ and $C_{12}$ components.

## 6. Results

### 6.1. Magnetization Measurements

**Figure 4:**
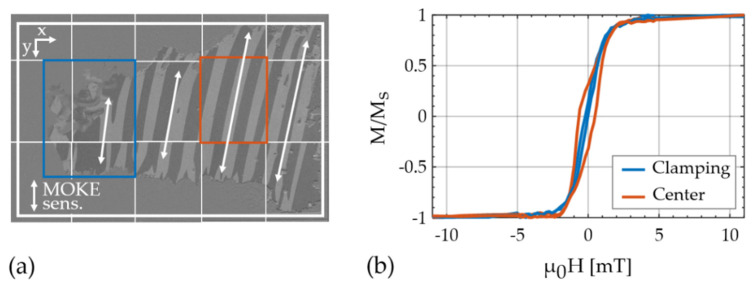
(**a**) Magneto-optical Kerr effect microscopy image of the analyzed structure, demagnetized along the x axis and composed of a series of different images. The region of the magnetic layer is marked by a white square and the approximated orientation of the magnetic easy axis is indicated with arrows at approximately −75°±5° relative to the x axis; (**b**) magnetization curve close to the clamping and in the center of the magnetic film. The two evaluated regions are indicated with squares in (**a**).

Magneto–optical Kerr effect (MOKE) microscopy [59] was used to analyze the magnetic multilayer. The picture in Figure 4a shows the rear side of the cantilever and is composed of a series of images. For each image, the magnetic multilayer was demagnetized along the x axis and the MOKE sensitivity axis was set along the y axis. The region of the magnetic multilayer is marked with a white frame and the estimated easy axis orientation is indicated with white arrows. In a large region around the left, top, and bottom edge, no magnetic response is visible. A comparison with light microscopy images reveals possibly corroded regions. They might have formed due to incomplete Cr-coverage at the edges. At the time, a particularly thin Cr-layer was deposited to ensure good magnetooptical contrast. Close to the clamping region (blue rectangle in Figure 4a), the layer is partially delaminated. Despite these nonidealities, the overall magnetic response in the magnetically active region is quite homogeneous. The average easy axis orientation is approximately $\varphi_{\mathrm{EA}}=-75{}^{\circ}\pm 5{}^{\circ}$ relative to the x axis. An effective uniaxial anisotropy energy density of $K=\left(1.2\;\pm 0.1\right)\;\mathrm{kJ}/m^{3}$ is estimated with the magnetoelastic model. We used the ballistic demagnetizing tensor [60] in the center of the film and assumed $\sigma_{j}=0$. A representative magnetization curve of the center region, recorded along the x axis, is shown in Figure 4b, and compared with one recorded at the clamping region. The difference between these curves indicates a different alignment of the effective anisotropy. However, due to the magnetic multilayer structure and the partial delamination additional effects cannot be excluded. From previous investigations [49], we expect that the deteriorated magnetic properties at the clamping especially reduce the resonance frequency detuning and the magnetic sensitivity of the first bending mode.

### 6.2. Electromechanical Properties

**Figure 5:**
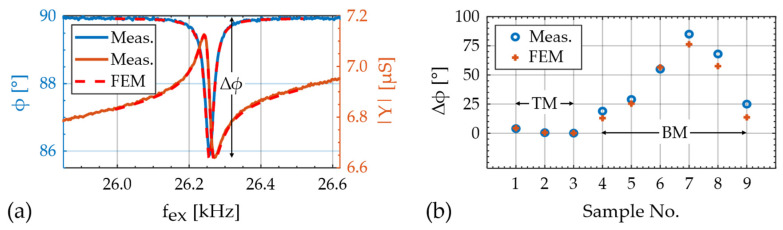
(**a**) Example comparison of measurement and FEM simulation of the sensor admittance around the first torsion mode (TM1), close to magnetic saturation at −10 mT; (**b**) comparison of measured and modeled maximum phase shift of the first three torsion modes (TM, samples 1–3) and the first and second bending modes (BMs, samples 4–9) with various electrode configurations. The BM measurements are published in Ref. [28].

To analyze the electromechanical properties of the sensor, the sensor admittance $Y\left(f_{\mathrm{ex}}\right)$ is measured over a large range of excitation frequencies $f_{\mathrm{ex}}$. Six resonance modes are characterized in detail by fitting a modified Butterworth van Dyke (mBvD) model (e.g., [61]) with the equivalent circuit configuration from [47] to the measurements. The resonance frequencies $f_{r}$ and quality factors are calculated from the mBvD parameters of each admittance curve and compared with the eigenfrequencies obtained from the finite element method (FEM) model. With this comparison, the eigenmodes are identified to be the first three bending modes (BM1–3) and the first three torsion modes (TM1–3). The FEM model was fitted to admittance measurements of the first torsion mode (TM1) close to magnetic saturation at $\mu_{0}H\;=-10\;\mathrm{mT}$. It matches the measurements very well as shown in Figure 5a. The material parameters match excellently with literature values. Details on the material parameters and on the geometry are given in Appendix C. A comparison of the measured resonance frequencies in magnetic saturation with the FEM simulations results in extremely small deviations <2% for all six modes (Appendix B).

The set of material parameters found is used to predict, and compare the impedance characteristic of other cantilever delta-E effect sensors published previously [28]. The sensors differ in their geometry from our torsion mode sensor. They were designed to excite the first and second bending mode with various electrode geometries. For the simulations, we used the same material parameters found for the torsion mode sensors but adjusted the geometry.

As a figure of merit for the electromechanical model, we compared the absolute difference $\Delta \phi =\phi_{\max}-\phi_{\min}$ of the phase angle ϕ of the electrical admittance Y. The simulation results are plotted in Figure 5b and compared with values of the torsion modes (Appendix B) measured here, and the bending mode from Ref. [28]. The TMs were measured close to magnetic saturation at $\mu_{0}H\;=-10\;\mathrm{mT}$ to reduce the influence of the delta-E effect. Slight deviations between the measurement and simulation might result from effectively different magnetoelectric coupling factors, e.g., due to the slightly different material parameters, geometric inaccuracies, or stress [62]. In conclusion, the model can estimate the electromechanical properties of the device and the effect of different electrode configurations well. For the application of magnetoelastic resonators as delta-E effect sensors, a high Δϕ and hence a high electrical sensitivity is desirable. In comparison to the bending modes, the Δϕ of the torsion modes is systematically smaller, which is also reflected in the electrical sensitivities. With $S_{Y,r}\approx 0.85\;\mathrm{mS}$ of TM1, the maximum relative electrical amplitude sensitivity $S_{Y,r}\approx 5.8\;\mathrm{mS}$ of sample No. 7 (BM2) [28] is almost a factor of seven larger, despite a similar quality factor. Hence, the large factor potentially gained in the magnetic sensitivity from utilizing the C_66_ component can be diminished by a reduced electrical sensitivity.

Additional simulations show that further optimization of the electrode design and reduction in the parasitic capacity from bond pads and wires could improve Δϕ of TM1 to Δϕ=10°. Alternatively, the parasitic effect of the sensor capacitance could be neutralized with additional electronics to utilize the phase-modulated signal for magnetic field detection [48]. A further improvement by a factor of two could be obtained by exciting both electrodes $E_{1}$ and $E_{2}$, phase shifted by 180°. Additionally, alternative piezoelectric materials with larger piezoelectric coefficients, such as AlScN [63,64,65] could increase the electrical sensitivity significantly and result in Δϕ comparable with bending modes.

### 6.3. Delta-E Effect and Sensitivities

**Figure 6:**
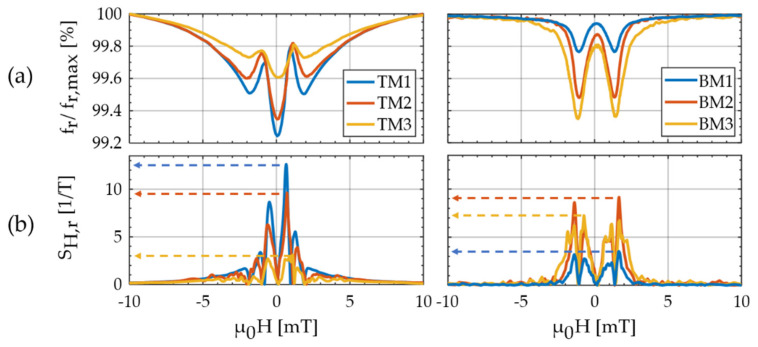
Measurements of the resonance frequency $f_{r}$ as a function of the applied magnetic flux density $\mu_{0}H$ along the long cantilever axis (x axis) starting at $\mu_{0}H=-10\;\mathrm{mT}$: (**a**) normalized resonance frequency $f_{r}/f_{r,\max}$ of the first three torsion modes (TMs) (left) and the first three transversal bending modes (BMs) (right). The maximum resonance frequencies $f_{r,\max}$ of the TMs are 26.256, 87.478, 175.150 kHz, and of the BMs: 7.649, 47.182, 121.400 kHz; (**b**) relative magnetic sensitivities $S_{H,r}=S_{H}/f_{r,\max}$ calculated from the data in (**a**) with Equation (1).

The $f_{r}\left(H\right)$ plots extracted from the modified Butterworth van Dyke (mBvD) fits of the first three bending modes (BM1–3) are shown in Figure 6a (right). They are normalized to $f_{r,\max}\Delta f_{r}\left(-10\;\mathrm{mT}\right)$ and have a respective minimum resonance frequency $f_{r,\min}$. As a measure for the maximum resonance frequency detuning, we defined the normalized resonance frequency change $≔f_{r}≔\left(f_{r,\max}-f_{r,\min}\right)/f_{r,\max}$. All three curves are w-shaped and $\Delta f_{r}$ increases with increasing mode number. This effect was reported previously and explained with a strong weighting of the magnetic properties at the clamping in BM1 [49]. Here, the difference between the BM1 and BM2 is significantly larger, which is consistent with the deteriorated material around the clamping region, visible in the magneto–optical Kerr effect microscopy (MOKE) images (Figure 4a). Correspondingly, the relative magnetic sensitivity $S_{H,r}\approx 3.5\;T^{-1}$ is smallest in BM1 and increases up to $S_{H,r}\approx 9\;T^{-1}$ in BM2.

The normalized $f_{r}\left(H\right)$ plots of the torsion modes (TMs) and their corresponding $S_{H,r}$ are shown in Figure 6 (left). Although the sample is close to magnetic saturation at $\mu_{0}H=-10\;\mathrm{mT}$, all three $f_{r}\left(H\right)$ curves still exhibit a non-zero slope as expected from the presented theory. The three $f_{r}\left(H\right)$ curves have a global minimum around H = 0, two local minima at around ±2 mT, and two local maxima at about ±1 mT. With an increasing mode number, the local maxima are almost unaffected, whereas $\Delta f_{r}$ significantly decreases. Consequently, the maximum $S_{H,r}$ also decrease with the increasing mode number, here from $S_{H,r}=12.6{\;T}^{-1}$ in TM1 to $S_{H,r}=3.0{\;T}^{-1}$ in TM3. The trend is notably opposed to the corresponding behavior of the bending modes and will be analyzed and explained in detail in the next section using the magnetoelastic and electromechanical models. Overall, the magnetic sensitivities are in the range of $\approx 10\;T^{-1}$ also measured with other magnetoelastic resonators in bending and bulk resonance modes [22,49]. At first glance, the similarity of BM and TM in $S_{H,r}\propto \partial_{H}C_{ij}$, might contradict the magnetoelastic model results in Figure 3c. To resolve this and explain the dependency of the torsion modes on the mode number, the second factor $\partial_{C}f_{r,ij}$ that contributes to $S_{H,r}$ must be considered.

### 6.4. Resonance Frequency Simulations

**Figure 7:**
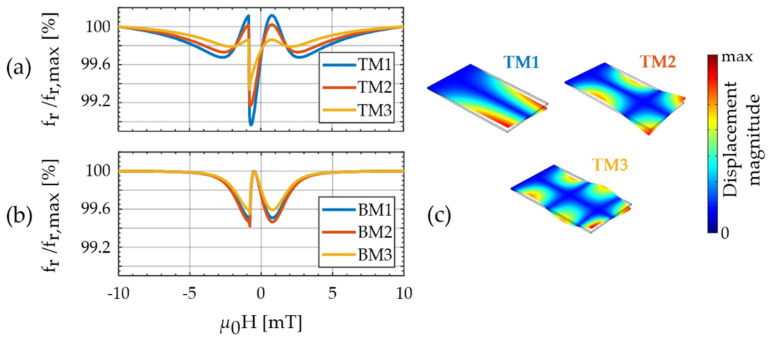
(**a**) Simulated resonance frequency $f_{r}$ normalized to its maximum, as a function of the magnetic bias flux density $\mu_{0}H$ for the first three torsional modes (TMs); and (**b**) the first three bending modes (BMs); and (**c**) the magnitude of the displacement vector of the first three torsion modes plotted and calculated with ${\mathrm{COMSOL}}^{®}$ Multiphysics v. 5.3a (COMSOL AB, Stockholm, Sweden).

In the following, we use the stiffness tensor components from the magnetic model as input in the finite element method (FEM) model to describe and analyze the frequency detuning and the magnetic sensitivity of the bending and torsion modes measured before. The demagnetizing tensor is approximated with the ballistic demagnetizing tensor in the center of the magnetic layer [60]. Consistently with the measurements the easy axis angle is set to $\varphi_{\mathrm{EA}}=-75{}^{\circ}$ and the effective anisotropy energy density constant to $K=1.2\;\mathrm{kJ}/m^{3}$, assuming $\sigma_{j}=0$. Results for the normalized resonance frequencies $f_{r}\left(H\right)$ of the torsion modes are shown in Figure 7a and of the bending modes in Figure 7b. Despite the simplifying assumptions, a striking similarity with the measurements is apparent. All simulated torsion mode (TM) curves in Figure 7a exhibit two local maxima around one global minimum. Due to the single-domain hysteresis, the local minimum is shifted slightly leftwards away from $\mu_{0}H=0$. The frequency difference between the local maxima and the global minimum decreases significantly with increasing mode number, as also observed in the measurements.

Within the model, this phenomenon can be explained with the mode shapes of the higher torsion modes (Figure 7c). Due to the multiple twisting of the cantilever in higher modes, the resonance nodes are closer together. This results in an increasing contribution of the stiffness tensor components *C*_11_ and *C*_22_ to $f_{r}$ relative to the *C*_66_ component. Quantitatively, we can explain the contribution of *C_ij_* to $f_{r}$ with the 11- and the 66-components of the normalized frequency factors $\partial_{C}f_{r,ij}$ (Equation (3)). They are estimated with the FEM model and summarized in Table 1. Whereas $\partial_{C}f_{r,11}$ increases by almost a factor of three, $\partial_{C}f_{r,66}$ shows the opposite trend and decreases by a factor of approximately two, from TM1 to TM3. Because the minima and maxima of *C*_11_ and *C*_66_ occur at similar magnetic bias fields they increasingly compensate each other in higher torsion modes. This causes similar magnetic sensitivities of TMs and BMs in our sensor, although $\partial_{H}C_{66,\max}>\partial_{H}C_{11,\max}$ in Figure 3c. If the delta-E effect of *C*_66_ is to be utilized, consequently, the first torsion mode is preferable to higher modes.

In contrast to the measured bending mode curves (Figure 6), the corresponding modeled curves (Figure 7b) are almost independent of the mode number. Consistently, the $\partial_{C}f_{r,11}$ of the BMs are approximately constant with the mode number. The other frequency factor $\partial_{C}f_{r,12}$ is very small and $\partial_{C}f_{r,66}\approx 0$. A different effect dominates the mode dependency observed in the measured bending modes. This corroborates the hypothesis stated earlier in Section 6.3 that the reduced maximum normalized resonance frequency change $\Delta f_{r}$ (as defined in Section 6.3) of BM1 is likely caused by the deteriorated magnetic layer present around the clamping (Figure 4a).

As shown earlier in Figure 3a, the minima of *C*_11_(*H*) occur at the same magnetic bias fields as the maxima of *C*_12_(*H*). Whereas *C*_11_(*H*) softens upon the application of a magnetic bias field, *C*_12_(*H*) increases. However, upon application of a magnetic field, they both reduce the resonance frequency of bending modes. Consequently, their corresponding frequency factors have opposite signs and $\partial_{C}f_{r,12}<0$.

## 7. Summary and Conclusions

In summary, we provide an experimental and theoretical study on the delta-E effect, the normalized resonance frequency change $\Delta f_{r}$ (defined in Section 6.3) and the sensitivity of first and higher-order bending modes (BMs) and torsion modes (TMs). The study was conducted on a magnetoelectric thin-film cantilever with a soft magnetic FeCoSiB–Cr multilayer and an electrode design that enables the excitation of various resonance modes. A general expression was developed that permits the detailed analysis of the magnetic sensitivity of arbitrary resonance modes. An electromechanical finite element method model was set up to describe the resonator and the electrical sensitivity. It was combined with a magnetoelastic macrospin model to include the tensor of the linearized delta-E effect for isotropic magnetostriction in the approximation of negligible magnetostrictive self-energy. The models are valid for moderately high-operation frequencies, where electrodynamic and magnetodynamic effects can be omitted.

The delta-E effect model is discussed in detail for here the most relevant components *C*_66_, *C*_11,_ and *C*_12_ of the magnetic field-dependent stiffness tensor. Simulation results imply that the *C*_66_ component potentially offers a ten-fold higher contribution to the magnetic sensitivity than the *C*_11_ component. With an increasing tilt of the magnetic easy axis, this factor reduces to approximately four at an easy axis angle aligned at 80° relative to the long axis of the cantilever. However, the measurements and simulations of the current design confirm that the TMs exhibit a systematically smaller electromechanical response compared to BMs, which can significantly diminish the potential gain in sensitivity. Possible ways of improvement are sketched out. From simulated and measured resonance frequency curves $f_{r}\left(H\right)$ we found that the maximum normalized resonance frequency change $\Delta f_{r}$ and the magnetic sensitivity of TMs reduce with the increasing mode number due to the increasing contribution of *C*_11_ to the resonance frequency. Hence, the dependency of TMs on the mode number is opposite to the one observed for BMs and caused by a different mechanism.

In conclusion, the delta-E effect of the *C*_66_ component shows the promising potential of significantly increasing the magnetic sensitivity and the maximum normalized resonance frequency change $\Delta f_{r}$. However, the efficient electrical excitation of TMs remains challenging for achieving high electrical sensitivity. Generally, the results imply that the delta-E effect of different *C_ij_* can have opposite effects on $\Delta f_{r}$, depending on the resonance mode. This was demonstrated in the example of torsion modes. Because the contribution of *C*_11_ increases with the torsion mode number, the first-order torsion mode shows the highest magnetic sensitivity. In addition to fundamental insights on the delta-E effect in higher resonance modes, a model for the electrical and the magnetic sensitivity was presented. The results are not only relevant for the development of magnetoelastic magnetic field sensors, but also for frequency tunable devices based on the delta-E effect.

## Figures and Tables

**Table 1:** Normalized frequency factors $\partial_{C}f_{r,11}$ and $\partial_{C}f_{r,66}$ (Equation (3)) of the first three torsion modes (TMs) and bending modes (BMs), calculated with the electromechanical finite element model.

Resonance Mode	TM1	TM2	TM3	BM1	BM2	BM3
$\partial_{C}f_{r,11}$	0.010	0.017	0.029	0.060	0.056	0.052
$\partial_{C}f_{r,66}$	0.034	0.026	0.016	0	0	0
$\partial_{C}f_{r,12}$	0	0	0	−0.003	−0.006	−0.001

## Data Availability

Not applicable.

## Appendix A. Magnetoelastic Model

### A.1. Definition of Vectors

In the following, a detailed definition of all vectors is given in the spherical coordinate system. All polar angles are denoted by θ and all azimuthal angles by φ, with an index if it is not the angles of the reduced magnetization. The reduced magnetization vector is denoted as

(A1) $$\bar{m}=\;{\left[\begin{array}{ccc} \cos\varphi \sin\theta & \sin\varphi \sin\theta & \cos\theta \end{array}\right]}^{T},$$

The easy axis unit vector $\bar{EA}$ is given by

(A2) $$\bar{EA}=\;{\left[\begin{array}{ccc} \cos\varphi_{\mathrm{EA}}\sin\theta_{\mathrm{EA}} & \sin\varphi_{\mathrm{EA}}\sin\theta_{\mathrm{EA}} & \cos\theta_{\mathrm{EA}} \end{array}\right]}^{T}.$$

The vector $\bar{H}$ of the external applied field and ${\bar{H}}_{d}$ of the demagnetizing field are given by

(A3) $$\bar{H}=H\;{\left[\begin{array}{ccc} \cos\varphi_{H}\sin\theta_{H} & \sin\varphi_{H}\sin\theta_{H} & \cos\theta_{H} \end{array}\right]}^{T},$$

(A4) $${\bar{H}}_{d}=-M_{S}{\left[\begin{array}{ccc} D_{11}m_{1} & D_{22}m_{2} & D_{33}m_{3} \end{array}\right]}^{T}.$$

For all higher-order tensors, we use the Voigt notation. The magnetostriction tensor is then given by

(A5) $$\lambda =\frac{3}{2}\lambda_{s}\;{\left[\begin{array}{cccccc} m_{1}^{2}-\frac{1}{3} & m_{2}^{2}-\frac{1}{3} & m_{3}^{2}-\frac{1}{3} & 2m_{2}m_{3} & 2m_{1}m_{3} & 2m_{1}m_{2} \end{array}\right]}^{T}.$$

### A.2. General Expression for $\Delta S_{ij}$

From the equilibrium conditions, one can write

(A6) $$\begin{array}{l} \Delta S_{ij}=\frac{\partial \lambda_{i}}{\partial \sigma_{j}}=\frac{\partial \lambda_{i}}{\partial \varphi }\frac{\partial \varphi }{\partial \sigma_{j}}+\frac{\partial \lambda_{i}}{\partial \theta }\frac{\partial \theta }{\partial \sigma_{j}}=\frac{\partial \lambda_{i}}{\partial \varphi }\left(-\frac{\partial u_{\varphi }}{\partial \sigma_{j}}/\frac{\partial u_{\varphi }}{\partial \varphi }\right)+\frac{\partial \lambda_{i}}{\partial \theta }\left(-\frac{\partial u_{\theta }}{\partial \sigma_{j}}/\frac{\partial u_{\theta }}{\partial \theta }\right)\\ ≔-\frac{\partial \lambda_{i}}{\partial \varphi }\frac{\partial u_{\varphi }}{\partial \sigma_{j}}\frac{1}{u_{\varphi \varphi }}-\frac{\partial \lambda_{i}}{\partial \theta }\frac{\partial u_{\theta }}{\partial \sigma_{j}}\frac{1}{u_{\theta \theta }}. \end{array}$$

### A.3. Derivatives of the Energy Density Functional

For in-plane magnetization (θ=π/2), easy axis $\left(\theta_{\mathrm{EA}}=\pi /2\right)$, and magnetic field $\left(\theta_{H}=\pi /2\right)$, the second-order derivatives of u (Equation (7)) are given by

(A7) $$\begin{array}{c} u_{\theta \theta }≔\frac{\partial^{2}u}{\partial \varphi^{2}}=3\lambda_{s}\left[\sigma_{11}\cos\left(2\varphi \right)-\sigma_{22}\cos\left(2\varphi \right)+2\sigma_{12}\sin\left(2\varphi \right)\right]+\mu_{0}M_{s}H\cos\left(\varphi -\varphi_{H}\right)+2K\cos\left(2\left[\varphi -\varphi_{\mathrm{EA}}\right]\right)\\ \;\;\;\;\;+\mu_{0}M_{s}^{2}\left(D_{22}-D_{11}\right)\cos\left(2\varphi \right) \end{array}$$

(A8) $$\begin{array}{c} u_{\theta \theta }≔\frac{\partial^{2}u}{\partial \theta^{2}}=3\lambda_{s}\left[\sigma_{11}\cos^{2}\varphi +\sigma_{22}\sin^{2}\varphi -\sigma_{33}+\sigma_{12}\sin\left(2\varphi \right)\right]+\mu_{0}M_{s}H\cos\left(\varphi -\varphi_{H}\right)+2K\cos^{2}\left(\varphi -\varphi_{\mathrm{EA}}\right)\\ \;\;\;\;\;-\mu_{0}M_{s}^{2}\left(D_{11}\cos^{2}\varphi +D_{22}\sin^{2}\varphi -D_{33}\right) \end{array}$$

## Appendix B. Resonance Frequencies and Sensitivities

A summary of the maximum measured resonance frequencies $f_{r,\max}$ at $\mu_{0}H=$ −10 mT is given in Table A1, together with the corresponding quality factor $Q_{\max}$ and the maximum magnetic sensitivities from Figure 6. The bending modes (BMs) and the torsion modes (TMs) were all measured using only electrode E1. Hence, the electrodes are not optimized for the bending modes. Additionally, the maximum quality factor of the BMs is a factor of three smaller than in TM1. Due to both factors, the electrical sensitivity of BMs is significantly smaller than that of TMs for our cantilever.

**Table A1:** Measured resonance frequencies $f_{r,\max}\;$ of the six modes analyzed and the quality factor $Q_{\max}$, both measured in magnetic saturation at $\mu_{0}H=$ −10 mT. The maximum magnetic sensitivity $S_{H}$ and the maximum relative magnetic sensitivity $S_{H,r}$ are obtained from Figure 6b. The maximum relative electrical sensitivities found are given by $S_{\phi ,r}$ and $S_{Y,r}$.

Mode	$f_{r,max}\;\left(kHz\right)$	$f_{r,model}\;\left(kHz\right)$	$Q_{max}$	$S_{H}\;\left(Hz/mT\right)$	$S_{H,r}\;\left(1/T\right)$	$S_{\phi ,r}\;(\text{°})$	$S_{Y,r}\;(µS)$
TM1	26.26	26.26	900	330	12.6	4780	850
TM2	87.48	88.20	700	837	9.5	59	27.5
TM3	175.15	179.20	280	542	3.0	115	95
BM1	7.65	7.80	300	26.7	3.5	2550	150
BM2	47.18	48.55	300	432	9.2	220	70
BM3	121.40	116.20	300	811	6.7	18	42

## Appendix C. Geometry and Material Parameters

### C.1. Geometry

The poly-Si cantilever was measured with an optical microscope to be L=3.12 mm long and W=2.15 mm wide. For the simulations, the length in the model was slightly adjusted within the measurement accuracy to 3.116 mm. The magnetostrictive layer was deposited directly at the clamping on the bottom side of the poly-Si cantilever and has a width of $W_{\mathrm{mag}}=2\;\mathrm{mm}$ and a length of $L_{\mathrm{mag}}=3.05\;\mathrm{mm}$. The AlN layer is of the same geometry but deposited on top of the poly-Si. The electrodes $E_{1}\;\mathrm{and}$ $E_{2}$ are positioned at the clamping on top of the AlN layer and the left and right edge, respectively. An additional parallel capacitance of $C_{0}=17.7\;\mathrm{pF}$ is used. It is consistent with the area of the bond pads, the conduction lines, and the relative electrical permittivity used for the AlN.

### C.2. Substrate (Poly-Si)

For the poly-silicon substrate, we use isotropic material parameters, with Young’s modulus $E_{\mathrm{Si}}=160\;\mathrm{GPa}$ [66,67] a Poisson’s ratio of $v_{\mathrm{Si}}=0.22$ [67] and a mass density of $\rho_{\mathrm{Si}}=2300\;\mathrm{kg}/m^{3}$ [68].

### C.3. Magnetic Material (FeCoSiB)

The mass density of FeCoSiB was experimentally determined by estimating the volume with profilometer measurements and the mass with a microbalance. The measurements were performed on a 6-inch wafer with a mean FeCoSiB layer thickness of approximately 1.5 µm. A density of $\rho_{\mathrm{FeCoSiB}}=\left(7870\pm 1350\right)\;\mathrm{kg}/m^{3}$ was obtained. For the simulation, a corresponding mass density of $\rho_{\mathrm{FeCoSiB}}=7700\;\mathrm{kg}/m^{3}$ was used. For the stiffness tensor of the mechanically isotropic magnetic film, we use:

(A9) $$C_{m,ij}=\left[\begin{array}{cccccc} C_{m,11} & C_{m,12} & C_{m,12} & 0 & 0 & 0\\ C_{m,12} & C_{m,11} & C_{m,12} & 0 & 0 & 0\\ C_{m,12} & C_{m,12} & C_{m,11} & 0 & 0 & 0\\ 0 & 0 & 0 & C_{m,44} & 0 & 0\\ 0 & 0 & 0 & 0 & C_{m,55} & 0\\ 0 & 0 & 0 & 0 & 0 & C_{m,66} \end{array}\right]$$

Using a Young’s modulus of E = 150 GPa and a Poisson’s ratio of v = 0.3, both at fixed magnetization, the non-zero components of the fixed magnetization stiffness tensor are:

(A10) $$C_{m,11}=C_{m,22}=C_{m,33}=\frac{E\left(1-v\right)}{\left(1+v\right)\left(1-2v\right)}=201.92\;\mathrm{GPa}$$

(A11) $$C_{m,12}=C_{m,13}=C_{m,23}=\frac{E\left(v\right)}{\left(1+v\right)\left(1-2v\right)}=86.54\;\mathrm{GPa}$$

(A12) $$C_{m,66}=C_{m,55}=C_{m,44}=\frac{E}{2\left(1+v\right)}=57.69\;\mathrm{GPa}$$

For the magnetoelastic simulations, we use a saturation magnetic flux density of $\mu_{0}M_{s}=1.5\;T$ [17] and saturation magnetostriction of $\lambda_{s}=35\;\mathrm{ppm}$ [17].

### C.4. Piezoelectric Material (AlN)

For the stiffness matrix $C_{\mathrm{AlN}}$ and the piezoelectric stress-charge coefficient tensor d we use values based on ab initio calculations [69]. Those tend to overestimate d and are here slightly adjusted. The stiffness tensor is:

(A13) $$C_{\mathrm{AlN}}=\left[\begin{array}{cccccc} 410.2 & 142.2 & 110.1 & 0 & 0 & 0\\ 142.2 & 410.2 & 110.1 & 0 & 0 & 0\\ 110.1 & 110.1 & 385.0 & 0 & 0 & 0\\ 0 & 0 & 0 & 122.9 & 0 & 0\\ 0 & 0 & 0 & 0 & 122.9 & 0\\ 0 & 0 & 0 & 0 & 0 & 134.0 \end{array}\right]\;\mathrm{GPa}\;$$

The piezoelectric coupling tensor $c_{e}=dC_{\mathrm{AlN}}$ results as

(A14) $$c_{e}=\left[\begin{array}{cccccc} 0 & 0 & 0 & 0 & -0.27828 & 0\\ 0 & 0 & 0 & -0.27828 & 0 & 0\\ -0.4496 & -0.4496 & 1.41 & 0 & 0 & 0 \end{array}\right]\frac{\mathrm{pC}}{m^{2}}.$$

For the density, we use $\rho_{\mathrm{AlN}}=3300\mathrm{kg}/m^{3}$ and for the electrical permittivity $\varepsilon_{\mathrm{el}}=\varepsilon_{r}\varepsilon_{0}$ with the electrical vacuum permittivity $\varepsilon_{0}$ and the relative electrical permittivity $\varepsilon_{r}$, given by

(A15) $$\varepsilon_{r}=\left[\begin{array}{ccc} 9.2081 & 0 & 0\\ 0 & 9.2081 & 0\\ 0 & 0 & 10.1192 \end{array}\right]$$
